# Supplementary material for: Reassessing the substrate specificities of the major Staphylococcus aureus peptidoglycan hydrolases lysostaphin and LytM
Source: eLife. 2024 Nov 4;13:RP93673. doi: 10.7554/eLife.93673 (PMC11534333; doi:10.7554/eLife.93673)
Supplement: Supplementary file 1. [file elife-93673-supp1.docx]

**Supplementary File 1.**

**Synthetic PG fragments used in this study**

|  | **Description** | **PG fragment** |
| --- | --- | --- |
| 1 | pentaGly (pGly) | H_2_N-GGGGG-COOH |
| 2 | pGly N-term linked to D-Ala-Lys | H_2_N-KDA-GGGGG-COOH |
| 3 | pGly N-term linked to tetrapeptide stem | H_2_N-ADiQKDA-GGGGG-COOH |
| 4 | pGly C-term linked to Nζ of Lys tetrapeptide stem | H_2_N-ADiQK(Nζ-GGGGG-NH_2_)DA-COOH |
| 5 | pGly C-term linked to Nζ of Lys in pentapeptide stem | H_2_N-ADiQK(Nζ-GGGGG-NH_2_)DADA-COOH |
| 6 | pGly C-term linked to Nζ of Lys in tetrapeptide stem and N-term to D-Ala-Lys | H_2_N-ADiQK(Nζ-GGGGG-DAK-NH_2_)DA-COOH |
| 7 | pGly C-term linked to Nζ of Lys in pentapeptide stem and N-term to tetrapeptide stem | H_2_N-ADiQK(Nζ-GGGGG-DAKDiQA-NH_2_)DADA-COOH |
| 8 | triGly C-term linked to Nζ of Lys in pentapeptide stem and N-term to tetrapeptide stem | H_2_N-ADiQK(Nζ-GGG-DAKDiQA-NH_2_)DADA-COOH |
| 9 | monoGly C-term linked to Nζ of Lys in pentapeptide stem and N-term to tetrapeptide stem | H_2_N-ADiQK(Nζ-G-DAKDiQA-NH_2_)DADA-COOH |
| 10 | GSGGG N-term linked to D-Ala-L-Lys | H_2_N-KDA-GSGGG-COOH |
| 11 | GGSGG N-term linked to D-Ala-L-Lys | H_2_N-KDA-GGSGG-COOH |
| 12 | tetraGly N-term linked to D-Ala-L-Lys | H_2_N-KDA-GGGG-COOH |
| 13 | triGly N-term linked to D-Ala-L-Lys | H_2_N-KDA-GGG-COOH |
| 14 | diGly N-term linked to D-Ala-L-Lys | H_2_N-KDA-GG-COOH |
| 15 | pGly N-term linked to L-Ala-L-Lys | H_2_N-KA-GGGGG-COOH |
